# Linking FOXM1 and PD-L1 to CDK4/6-MEK targeted therapy resistance in malignant peripheral nerve sheath tumors

**DOI:** 10.18632/oncotarget.28650

**Published:** 2024-09-30

**Authors:** Joshua J. Lingo, Ellen Voigt, Dawn E. Quelle

**Affiliations:** ^1^Cancer Biology Graduate Program, University of Iowa, Iowa City, IA 52242, USA; ^2^Holden Comprehensive Cancer Center, University of Iowa, Iowa City, IA 52242, USA; ^3^Medical Scientist Training Program, University of Iowa, Iowa City, IA 52242, USA; ^4^Department of Neuroscience and Pharmacology, University of Iowa, Iowa City, IA 52242, USA; ^5^Department of Pathology, University of Iowa, Iowa City, IA 52242, USA

**Keywords:** MPNST, FOXM1, PD-L1, CDK4/6-MEK targeting, therapy resistance

## Abstract

Malignant peripheral nerve sheath tumors (MPNSTs) are aggressive, Ras-driven sarcomas characterized by loss of the *NF1* tumor suppressor gene and hyperactivation of MEK and CDK4/6 kinases. MPNSTs lack effective therapies. We recently demonstrated remarkable efficacy of dual CDK4/6-MEK inhibition in mice with *de novo* MPNSTs, which was heightened by combined targeting of the immune checkpoint protein, PD-L1. The triple combination therapy targeting CDK4/6, MEK, and PD-L1 led to extended MPNST regression and improved survival, although most tumors eventually acquired drug resistance. Here, we consider the immune activation phenotype caused by CDK4/6-MEK inhibition in MPNSTs that uniquely involved intratumoral plasma cell accumulation. We discuss how PD-L1 and FOXM1, a tumor-promoting transcription factor, are functionally linked and may be key mediators of resistance to CDK4/6-MEK targeted therapies. Finally, the role of FOXM1 in suppressing anti-tumor immunity and potentially thwarting immune-based therapies is considered. We suggest that future therapeutic strategies targeting the oncogenic network of CDK4/6, MEK, PD-L1, and FOXM1 represent exciting future treatment options for MPNST patients.

Malignant peripheral nerve sheath tumors (MPNSTs) are deadly sarcomas of the myelinating nerve sheath. They arise sporadically in 50% of all patients but are highly associated with a tumor predisposition syndrome, called Neurofibromatosis Type I (NF1). In NF1 patients, MPNSTs are the leading cause of mortality and they arise from the malignant transformation of benign plexiform neurofibromas (pNFs) [1]. As depicted in Figure 1, inactivation of the *NF1* tumor suppressor gene is the initiating event in all MPNSTs, which increases RAS-MAPK signaling and MEK activation [2]. The next most frequent alteration (in nearly 90% of MPNSTs) is loss of the *CDKN2A* tumor suppressor, which results in hyperactivation of oncogenic Cyclin-Dependent Kinases 4 and 6 (CDK4/6) [2–7].

**Figure 1:**
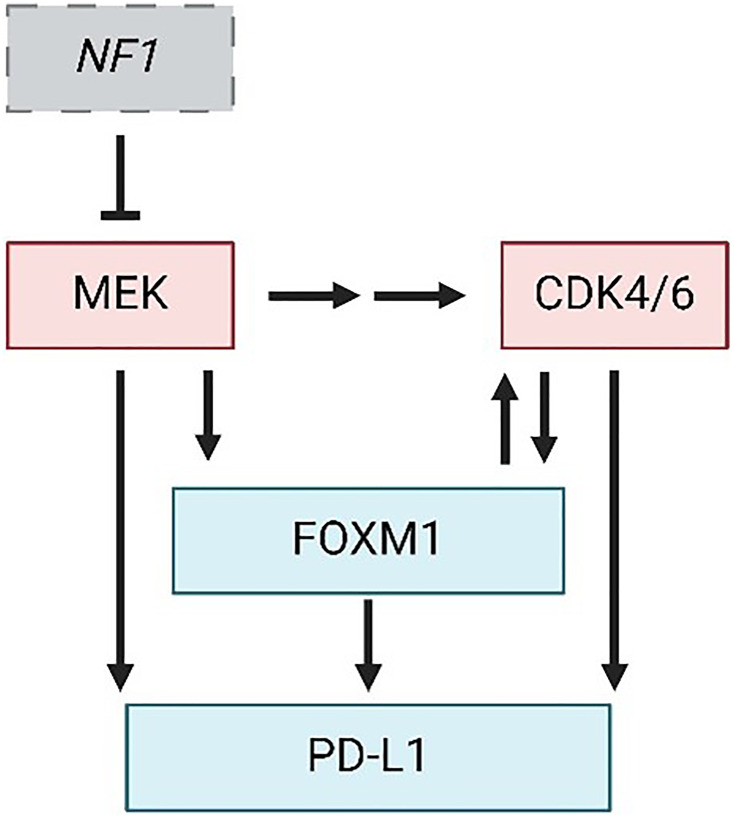
Central mechanisms of MPNST progression and therapy resistance. Pathway diagram depicting *NF1* loss and hyperactivation of MEK and CDK4/6 kinases as defining events in MPNST formation. FOXM1 and PD-L1 are downstream effectors of MEK and CDK4/6 whose upregulation likely mediates resistance to inhibitors of those kinases. Perpendicular bar, inhibition; Arrow, activation. Figure made with https://www.biorender.com/.

MPNSTs unfortunately lack effective therapies. The only curative treatment is complete surgical resection with clean, negative margins, but this is often not possible due to tumor size, location, and/or presence of metastatic disease [1]. Radiation and chemotherapy have limited efficacy and significant toxicity, making them poor options for treatment. To date, there still exists limited clinical trial data for immune checkpoint blockade (ICB) therapy in MPNST, although in most other types of sarcomas treatment with ICB agents failed to meet RECIST criteria [8]. Uniformly, the field has recognized a need for improved, more directed therapeutics to treat MPNSTs and many recently identified drivers of the disease are considered relevant clinical targets. For this commentary, we will discuss evidence supporting combination therapies against CDK4/6 and MEK for treating MPNSTs and their contribution toward anti-tumor immunity. We will also consider likely mediators of acquired resistance to such therapies, particularly Forkhead box protein M1 (FOXM1) and programmed death-ligand 1 (PD-L1) (Figure 1), whose simultaneous inhibition may be needed to achieve sustained, and possibly curative, anti-tumor activity.

We previously reported that *de novo* MPNSTs initiated by *Nf1* and *Cdkn2a* inactivation in the sciatic nerve are sensitive to CDK4/6 inhibitors; however, drug resistance emerged rapidly in all tumors [6]. MEK is well known to promote resistance to CDK4/6 inhibitors by enhancing the transcription of CDK4 and CDK6 as well as their regulatory partners, the D type cyclins [9, 10]. As such, other groups explored combinations of CDK4/6 and MEK inhibitors in Ras-driven lung [11] and pancreatic cancers [12], where remarkable success of the combination was observed [13]. Those successes, as well as CDK4/6 and MEK hyperactivation in patient MPNSTs, propelled our examination of combined CDK4/6 and MEK inhibition in our *de novo* MPNST model [14]. As anticipated, single agent CDK4/6 or MEK inhibitors provided a modest survival benefit reflecting slowed tumor growth whereas dual CDK4/6-MEK inhibition induced tumor regression and greatly extended survival. Shrinkage of the tumors by CDK4/6-MEK targeting was significant in both magnitude and duration. Nonetheless, despite early tumor regression the effects were transient as 100% of tumors eventually regrew due to acquired drug resistance.

To determine the mechanisms underlying MPNST response to CDK4/6-MEK inhibitor therapy, gene expression profiling of drug sensitive versus drug resistant tumors was performed [14]. The most prominent difference was an immune activation profile in drug sensitive tumors that was lost in resistant tumors. Activation of anti-tumor immunity was expected in sensitive tumors because they regressed with drug treatment. Moreover, combined CDK4/6-MEK therapy was previously shown to activate CD8+ T cell or natural killer (NK) cell-mediated immune responses in other tumor types [11, 12]. What was surprising in the MPNST analyses, however, was a dominant signature for B and/or plasma cell activation within the drug sensitive tumor [14]. Histopathological evaluation of the tumors verified an accumulation of plasma cells, not B cells, in tumors that shrunk upon CDK4/6-MEK inhibition. Enhanced clustering of CD8+ T cells, an indicator of their activation, was also observed. Conversely, resistant tumors adopted a highly immunosuppressive environment enriched with M2 tumor-associated macrophages (TAMs) and elevated expression of PD-L1 in the tumor cells (Figure 2).

**Figure 2:**
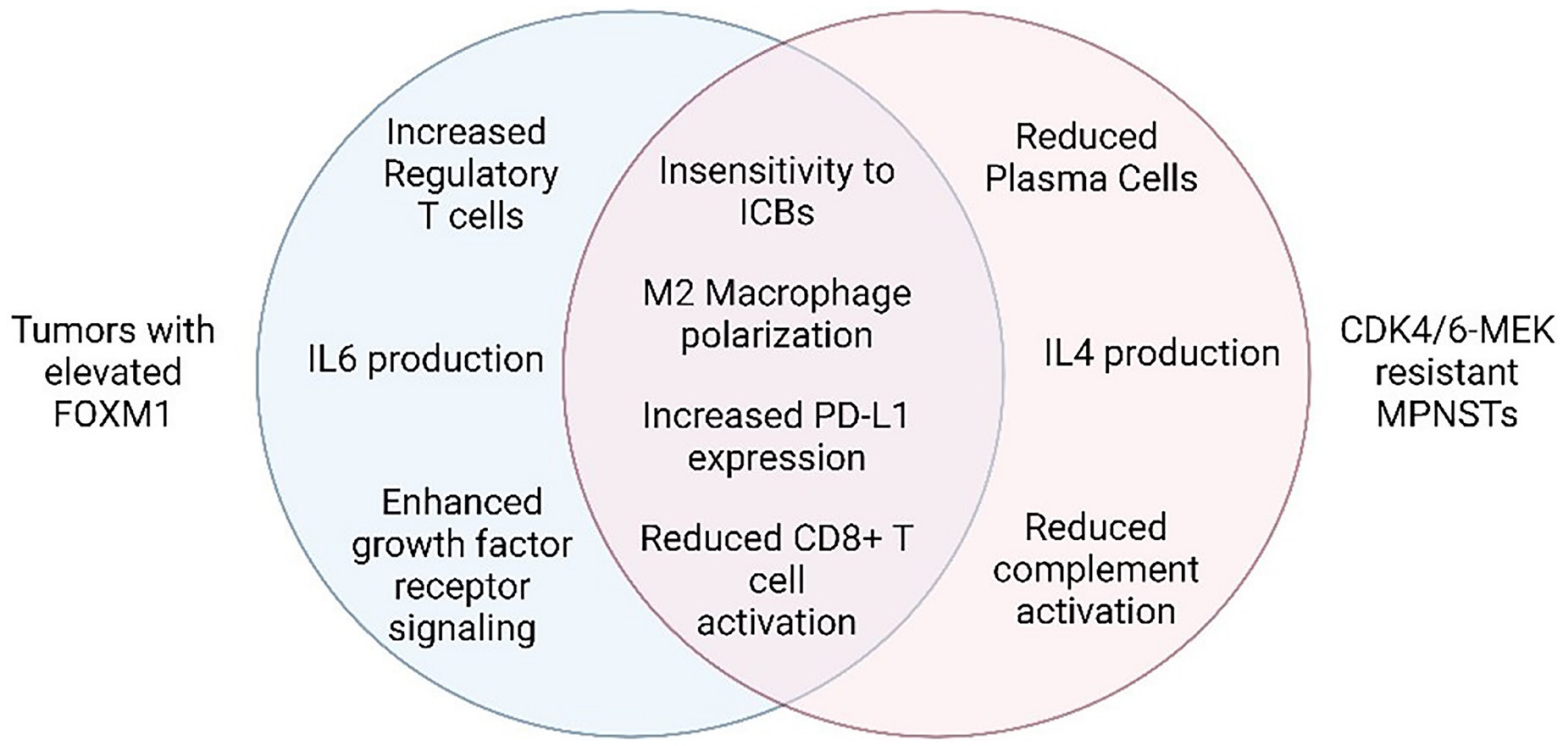
Hallmark features of CDK4/6 and MEK inhibitor resistance and FOXM1 elevation in tumors. Venn diagram indicating the shared (middle) and unique (left and right) features of each setting. Figure made with https://www.biorender.com/.

The plasma cell discovery was exciting because those immune cells are increasingly appreciated to connote enhanced patient survival and improved response to ICBs in many other types of human cancers [15–19]. Such associations have been seen in a few types of sarcoma, but so far analyses have not extended to include MPNSTs [15, 19]. Our mouse MPNST model findings predicted that CDK4/6-MEK inhibitor therapy would sensitize MPNSTs to ICB therapy, not only because of increased plasma cells but also given the upregulation of PD-L1 in drug resistant MPNSTs. That possibility was tested by combining PD-L1 antibody therapy with CDK4/6-MEK inhibition, which did indeed elicit impressive anti-tumor effects [14]. The triple combination not only led to sustained tumor regression and improved animal survival relative to individual drugs, but it was also curative (complete disease ablation) in 12% of mice. These results support the conclusion that PD-L1 upregulation promotes resistance to CDK4/6-MEK inhibition therapy. Moreover, we speculate that the best responders to therapy (those that were cured) may have had the highest accumulation of plasma cells since they are thought to be critical orchestrators of anti-tumor immunity [19, 20]. However, the necessity and role of plasma cells in mediating response to therapy and anti-tumor immunity, in any tumor type or treatment setting, remains to be tested.

Although CDK4/MEK/PD-L1 combination therapy proved highly effective in MPNSTs, the majority of tumors still eventually became resistant and resumed growth [14]. Given the increasing clinical use of inhibitors to CDK4/6, MEK, and PD-L1 in cancer patients, either as monotherapies or in combination strategies, finding new targets to combat resistance to these agents is of significant interest. Many potential mediators of resistance to CDK4/6 and MEK inhibitors have already been defined [10, 21]. High on that list may be the protein tyrosine phosphatase, SHP2, a positive regulator of many oncogenic signaling pathways that functionally interacts with RAS-MEK-ERK, CDK4/6-RB, and PD-1/PD-L1 pathways [22]. The Pratilas lab showed that SHP2 inhibitors act synergistically with MEK inhibitors [21] and more so with CDK4/6 inhibitors [23] to suppress MPNST growth. The excitement about dual CDK4/6-SHP2 inhibition has even led to clinical testing of ribociclib (CDK4/6 inhibitor) plus TNO155 (SHP2 inhibitor) in patients with NF1-deficient cancers (NCT04000529). The combination of CDK4/6-SHP2 inhibitors with ICB therapy would also merit testing as it may have greater potential for durable antitumor activity.

Another likely mediator of therapy resistance in CDK4/MEK/PD-L1 targeted MPNSTs is the FOXM1 oncoprotein [7]. As a transcription factor, FOXM1 controls the expression of numerous genes important for cellular proliferation, survival, and metastasis, making it a powerful and promising target in many human cancers [24]. It has been minimally studied in MPNSTs but, for several reasons, may play a key role in the disease and therapy resistance. First, FOXM1 expression is elevated in patient MPNSTs, along with CDK4, which prognosed worse survival [25]. In agreement, Aimaier et al demonstrated a direct role for FOXM1 in promoting MPNST pathogenesis through knockdown and overexpression studies [26] while we have discovered that FOXM1 expression and transcriptional activity are greatly increased as benign human precursor pNFs transform into MPNSTs (unpublished data). Second, FOXM1 is phosphorylated at many sites by CDK4/6 during the G1/S phase of the cell cycle and these modifications activate FOXM1 transcriptionally [27] (Figure 1). In turn, FOXM1 represses expression of FOXO1, a transcription factor, that normally promotes expression of the CDK inhibitors p16, p21, and p27 [28, 29]. Third, ERK1/2 can phosphorylate FOXM1 at sites that promote its translocation into the nucleus and increase its transcriptional activity, indicating that MEK inhibition (which acts upstream of ERK) could effectively prevent this interaction [30, 31]. Fourth, in breast cancer studies, CDK4/6 inhibitors synergized with novel compounds that target FOXM1 [32, 33]. Finally, FOXM1 has been shown to promote resistance to a broad range of therapies including DNA damaging agents [34, 35], radiation [36], and several targeted agents including inhibitors of CDK4/6 [10, 27], PI3K [10], and EGFR [37].

Notably, tumors with elevated FOXM1 share many features with MPNSTs that are resistant to CDK4/6-MEK inhibition, most of which reflect extra-tumoral changes that suppress anti-cancer immunity [38, 39] (Figure 2). For instance, FOXM1 directly binds to the PD-L1 promoter (*CD274* gene) to activate its transcription [40] (see Figure 1), which aligns with our observation of upregulated PD-L1 protein in MPNSTs that overcame CDK4/6 and MEK inhibition. In agreement, PROTAC degraders of FOXM1 protein, as well as knockdown of FOXM1, decrease PD-L1 expression [41]. As for specific effects of FOXM1 on immune cell populations, one study in esophageal adenocarcinoma suggested FOXM1 inhibits CD8+ T cell chemotaxis, tumor infiltration, and tumor cell killing, in part through regulation of Th1 chemokine expression [42]. In cholangiocarcinoma, FOXM1 promoted FoxP3+ Treg cell tumor infiltration, thereby suppressing CD8+ T cell activity [43]. In lung adenocarcinoma, phosphorylated FOXM1 was shown to recruit monocytes and promote M2 macrophage polarization when tumor cells were co-cultured with macrophages [44]. MPNSTs resistant to CDK4/6-MEK inhibition likewise displayed reduced CD8+ T cell activation and increased M2 TAMs (Figure 2). FOXM1 can also prevent the maturation of bone marrow-derived dendritic cells in pancreatic ductal and colorectal adenocarcinomas [45]. Several of the pro-tumorigenic immune changes induced by FOXM1 in tumors reflect activated transcription of cytokine genes, such as *IL-6*, *IL1β*, and *CCL4* [38, 39]. Of note, heightened FOXM1 expression in osteosarcoma correlates with decreased response to immunotherapy [46], bolstering the possibility that inhibition of FOXM1 may be key to preventing ICB therapy resistance in our sarcoma model of MPNST.

In sum, the findings discussed herein suggest that FOXM1 inhibition may block or effectively delay acquired resistance of MPNSTs to sustained ICB immunotherapy and/or CDK4/6-MEK inhibition. There is in fact growing enthusiasm for targeting FOXM1 in cancer using newly developed FOXM1 inhibitors [7], particularly for controlling drug resistance as part of combination therapies [33]. More pre-clinical work is warranted to move those drugs into the clinic, but other potential mediators of resistance to CDK4/6-MEK inhibitors and ICB agents, such as SHP2 inhibitors, are already being tested clinically. Moving forward, this represents an exciting time of discovery that should guide better treatment options for patients with MPNSTs as well as other solid Ras-driven cancers.
